# Mortality and Early Feeding Behavior of Female Turkey Poults During the First Week of Life

**DOI:** 10.3389/fvets.2019.00129

**Published:** 2019-04-25

**Authors:** Colleen Roehrig, Stephanie Torrey

**Affiliations:** ^1^Campbell Centre for the Study of Animal Welfare, University of Guelph, Guelph, ON, Canada; ^2^Department of Animal Biosciences, University of Guelph, Guelph, ON, Canada

**Keywords:** poult, turkey, mortality, early feeding behavior, welfare

## Abstract

Turkey poults are susceptible to early mortality and poor initial feeding behavior for reasons that are not well-understood. This study was conducted to investigate the relationship between the development of turkey poult feeding behavior and early mortality, with a focus on the effect of biological age and diet. We hypothesized that increasing biological age would increase mortality, and that poults that had earlier feeding behavior would have decreased mortality. Nine hundred and sixty female turkey poults were randomly assigned to 24 pens (40 poults per pen). The study was conducted as a completely randomized block design with a factorial arrangement of two ages (early and standard hatch) and three diets (control, modified A, and modified Ca/P B, differing primarily in the Ca/P ratio and percent fines). The behavior during the first 24 h after placement of newly placed poults was examined. Growth performance and mortality throughout the first week of life was also measured to determine if early behavior had any impact on these variables over time. Behavior during the first 24 h was similar between the age treatments; both spent ~2.5% of the time drinking, 7% of the time feeding, 12% of the time active and 80% of the time inactive/resting. There was no effect of diet or age treatment on latency to feed or drink. Growth variables were not affected by biological age. However, there was a significant diet and biological age effect on bodyweight and mortality. Standard hatch poults fed a control diet were lighter than other poults at 7 d, while standard hatch poults fed the diet with the lowest Ca:P had the highest mortality. For all treatments, early mortality was primarily due to yolk sac infection, although >1% of placed poults died due to starvation. Findings of the present study indicate that, regardless of biological age or diet, poults established feeding behavior within the first 24 h and the majority of early poult mortality under these experimental conditions was due to factors other than starvation.

## Introduction

Many turkey poults may experience poor starting performance for reasons that are not well-understood. Mortality rates up to 6% within the first week of life have been reported for turkey flocks (1–3). Post-mortem examinations of some birds have shown that they had no feed in the digestive tract, leading to a condition called “Starve-Outs” (4). Additionally, a small percentage of birds are compromised and die or are culled from the flock when they fall onto their backs or sides and cannot right themselves, a condition commonly referred to as “flips” (2, 5). Both of these scenarios present obvious welfare concerns for the poults. Initial feeding behavior is suspected to be the primary contributor to these early mortality and performance problems for poults. Yet, the pattern of early feeding behavior for poults has not been well-defined in the literature. Previous research has focused on reducing early mortality through exogenous factors that were thought to stimulate feeding behavior. These exogenous factors include coloring the diet (6), changing the feed form (7), attracting the birds with lights (8), or sounds (5) or using a trainer bird (9). Although these methods attracted birds to the feeder, they generally did not have any effect on reducing mortality. Other dietary manipulations such as changing the nutrients or the feed texture have had some effect on young turkeys' growth and viability (3, 4, 10), although not always (11, 12). Yet, little attention has been paid to the effect of mineral concentrations and feed texture on the development of behavior in neonatal poults. Poultry may have a specific appetite and preference for some minerals, such as calcium (13), although what effect mineral concentrations, and in particular the ratio of calcium to phosphorus, has on the development of feeding is unknown. Higher proportions of fine particles in the diet may take longer for birds to consume (14), but whether proportion of fines impacts the development of feeding is also unknown.

Endogenous factors such as biological age (the age at time of hatch from time of egg placement), genetics, physiology of the digestive tract at hatch, sex and yolk sack size impact mortality, although their role in early feeding behavior of poults has received little attention. Turkey poult biological age at hatch can vary from 26.5 to 28.5 days, which results in some poults being in the hatcher 36–48 h longer than other poults. At hatch, the poult has ~6 to 9 g of yolk remaining in the peritoneal cavity (15), which serves as its only source of nutrients until placement. As the residual yolk at hatch accounts for only 10–12% of a poult's body weight (16), delayed access to feed and water can lead to dehydration (17) and changes to metabolic functioning (10). Moran (4) reported double the mortality rate for poults placed 24 h after hatch compared to those placed 6 h after hatch, and suggested starvation as the primary cause of these mortalities. Others have found that biological age plays a role in growth rates, with a longer time between hatch and placement leading to slower growth (3, 18). However, the role of biological age on feeding behavior in turkey poults has received little attention. In a pilot experiment, Panning (9) found that earlier hatched poults had a shorter latency to feed and drink than later hatched poults, although they were housed together and no mortality occurred during the 48 h experiment. Therefore, the objective of this study was to examine the relationships among biological age, the development of poult feeding behavior and mortality during the first week of life. We hypothesized that the behavior during the first 24 h after placement would differ between the age groups, resulting in a higher mortality rate in the early hatch compared to standard hatch group during the first week of life. This study was performed concurrent with a proprietary nutrition study assessing calcium and phosphorus levels in the starter diets. We hypothesized that the development of feeding behavior would not be influenced by diet.

## Materials and Methods

This study was approved by the University of Guelph Animal Care Committee, and conducted at the Shur-Gain Research Facility, Burford, Ontario in 2013.

### Study Design

This study was performed concurrent with a proprietary nutrition study assessing calcium and phosphorus levels in the starter diets. The study was designed as a completely randomized block design with a factorial arrangement of two biological ages and three starter diets. Pen was the experimental unit and there were 4 pens per treatment (*n* = 24 pens), using a 2 × 3 complete block design. Poults were divided into two biological age treatments, early and standard hatch, and pens were assigned to one of three dietary treatments. Biological age was defined as early or standard where early hatch birds were at least 24 h old at the time of removal from the hatcher and standard hatch group represented a random assortment of ages from the cohort hatch group.

### Birds, Housing, and Diet

Nine hundred sixty day old female turkey poults (Hybrid Convertor) were used for this study, with 40 poults per pen. Pen size was 1.8 × 3.7 m (6.01 birds/m^2^) and clean wood shavings were used for bedding. The room was heated by four forced air natural gas heaters and ventilated by ceiling air inlets on the south wall and wall ventilation fans on the north. Round metal pan feeders were used (one feeder per pen) providing 324 cm of total feeding space, with a rim height of 7 cm.

Pan feeders were placed on the floor for the first 3 d of study and then suspended after d3 to discourage roosting in the feed. There were three suspended cup and nipple drinkers per pen. Penning consisted of solid white plastic partitions from the floor to 30 cm of height, topped by metal wire panels to 200 cm of total pen height. The room was heated to 32°C prior to poult placement, following the research facility standard operating procedures. Each pen had a heat lamp suspended 60 cm above the bedding at the front half of the pen. The heat lamp remained on for the duration of the study. Average recorded temperature under the heat lamp was 36.3°C. Average pen temperature at bird level away from the heat lamp was 32.6°C. Temperature was reduced by 0.3°C per day until the end of the 7 d study.

The room was lit with dimmable incandescent bulbs. One hour prior to placement of poults, light level was measured using a light meter (cal-LIGHTv400, Cooke Corporation, New York, USA). Light intensity was measured in one pen per block and averaged. Under the heat lamps, light intensity averaged 267 lux, and was an average of 8 lux in the front and back of the pen. Lights remained on continuously for the first 24 h, and then the photoperiod was stepped down by 1 h/d to result in 20L:4D by d 4.

Diets were commercially produced (Yantzi Feed and Seed, Tavistock, Ontario, Canada) as turkey starter research diets and the texture was fine crumble. Feed was analyzed for nutrient content, durability and fines. Physical characteristics were similar between dietary treatments. Nutrient content differed slightly among the three diets (Table 1), with main differences in calcium and phosphorus levels and percent fines. Metabolizable energy was constant between diets at 10.8 MJ/kg. The control diet had a Ca/P ratio of 1.27. The first experimental diet (Modified A) had a Ca/P ratio of 1.30, with higher levels of both Ca and P compared to the control. The second experimental diet, (Modified B) had a Ca/P ratio of 1.22, with higher levels of both Ca and P compared to the control (Table 1). Feed was weighed into the feeders prior to the placement of poults and provided on an *ad libitum* basis throughout the study.

**Table 1:** Nutrient concentration and quality of the three starter diets used.

**Item (as fed)**	**Control**	**Modified A**	**Modified B**
Fat %	4.20	4.12	4.34
Ash %	6.71	6.59	6.73
Protein %	29.00	28.70	29.20
Crude fiber %	2.97	3.12	3.09
Calcium %	0.90	1.20	1.06
Available phosphorus %	0.71	0.92	0.87
Ca:P	1.27	1.30	1.22
Durability %	94.2	93.9	94.6
Fines %	25.9	19.7	20.7

*Metabolizable energy for each diet was 10.8 MJ/kg*.

### Hatchery and Placement

At the hatchery (Cold Springs Farm, Thamesford, Ontario) 24 h prior to removal from the hatcher, poults that had hatched were identified by placing a blue leg band (Kuhl Corporation, New Jersey, USA) on their right leg. The banded poults were placed back into the hatch basket with their conspecifics. These banded birds represented the early hatch group. On the morning of placement, all poults were infrared beak treated and toe processed as per the hatchery standard poult regimen and the banded poults were separated into labeled boxes for transport. Standard age poults were randomly selected from the available pool of non-banded poults.

Poults were shipped to the research facility in standard poult boxes (100 birds/box) and transit time was approximately 1 h. Upon arrival, the standard hatch poults were leg banded. Poults were then randomly selected from the appropriate age group hatchery boxes, individually weighed and placed into a holding box. This process was repeated for the 6 pens within a block. Once birds were randomly allocated to the 6 pens within the block, poults were placed into the appropriate pens. All birds in the block were placed simultaneously at the front of their respective pens. Allocation of birds to treatments was randomized, with treatments blocked within the room. Placement in pens occurred between 13:00 and 15:00 on the d 1.

### Behavior Observations

Prior to bird placement, video cameras were positioned to allow for video recording of activity in the pen and around the feeder. Videos were recorded onto a digital recording unit and transferred to an external hard drive at the end of the experiment. Recording were initiated prior to the bird placement in order to capture the initial feeding behavior. Latency to first feed and drink (in sec) were defined as the time at which the first poult in the pen was seen with its head at the feeder or drinker.

Behavior time budget was determined by instantaneous scan sampling the behavior of all poults within the pen every 10 min for the first 24 h after placement. Behaviors that were recorded were feeding (head at or in the feeder, pecking at the feeder), drinking (head in or at the drinker, pecking at the drinker), active (walking, preening, pecking at the litter, interacting with conspecifics) and inactive (resting, not visible on camera or other behaviors not previously defined). Data was reported as a percentage of time the behavior occupied of the total time budget, and then pooled into hourly values to give 24 measures per pen per behavior.

On days 2, 3, and 5 after placement, instantaneous scan sampling was performed every 10 min from 9:30 to 11:30 and 14:30 to 16:30, for a total of 13 observations per pen per period (morning or afternoon) per day. The percentage of poults performing feeding, drinking, active and inactive behavior at each scan within the morning or afternoon was averaged for each pen, to give a percentage of time the behavior occupied of the total time budget.

### Morbidity and Mortality

Physical observations of bird activity were made 4times per day (8:00, 12:00, 16:00, 20:00) for the first 4 d of the study in order to capture flip occurrences. After d 4, observations were made twice a day as per research facility standard operating procedure. When poults were identified as “Flips,” they were righted if possible and identified by marking the right foot with colored animal paint. In the event a bird was found flipped at a subsequent observation, it was marked on the left foot. Flips that were too weak to right themselves were removed from study and euthanized. Flip and mortality data was quantified into total occurrences per pen for the first 7 d of study. Cause of death was determined for all mortalities by a poultry veterinarian.

After d 7, the behavior portion of the study concluded, leg bands were removed and the birds continued on the concurrent nutritional study.

### Growth Performance Measures

Poults were weighed individually at 0, 3, and 7 d of age. Average bodyweight, variation within pen and average daily gain was determined on a pen by period basis. Feeders were removed from pens 2 h prior to weighing to minimize variability in gut fill and returned immediately after the bodyweights were measured to minimize the feed withdrawal period. Feed weight was taken to determine pen feed intake. Average daily feed intake, total feed intake and feed conversion ratio was determined on a pen by period basis.

### Statistical Analysis

Variation within treatment and variables was analyzed by Brown and Forsythe's test for Homogeneity. Performance data (bodyweight, average daily gain, average daily feed intake, feed conversion ratio, mortality and flip occurrences), 24 h behavior, behavior through d 5, and latency to feed and drink were analyzed using the Mixed procedure of SAS (SAS Institute, Cary, NC, USA). Biological age and diet treatments were considered as fixed effects and block was considered a random factor. Latency to feed and drink data was log transformed prior to statistical analysis. The 24 h behavior data was graphed to determine the appropriate grouping of data; 6 h time intervals were grouped and the data was then arcsine-square root transformed and averaged prior to analysis. This grouped behavior data was analyzed using orthogonal contrasts (Helmert) using SAS. Period 1 was 0–6 h after placement; period 2 was 7–12 h after placement; period 3 was 13–18 h after placement; period 4 was 19–24 h after placement. Results were considered significant when *P* ≤ 0.05.

## Results

### Initial Time Budget

**Figure 1:**
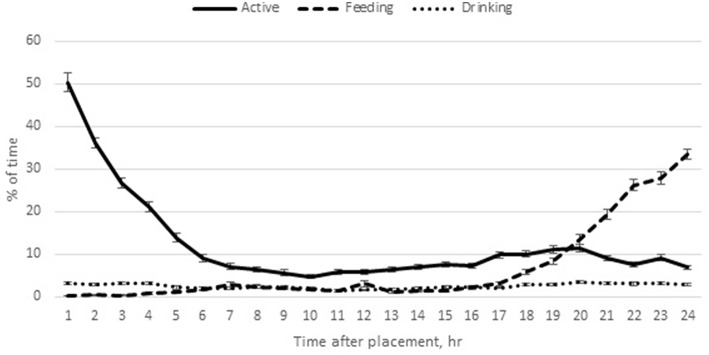
Mean percentage (± SE) of time poults spent active, feeding and drinking over the first 24 h after placement. Remaining time budget was spent inactive.

Feeding behavior during the first 24 h after placement was similar between the age treatments [*F*_(1, 18)_ = 0.67; *P* = 0.42] and dietary treatments [*F*_(2, 12.6)_ = 1.33; *P* = 0.30]. There was no interaction between biological age and dietary treatments [*F*_(2, 18)_ = 0.34; *P* = 0.72]. Over the course of the 24 h, poults spent 6.93 ± 0.22% of time feeding. There was a significant effect of time on feeding behavior [*F*_(3, 44)_ = 105.61; *P* = 0.0001]; within the first 6 h of placement, feeding behavior was rare; 0.80 ± 0.09% of time was spent feeding (Figure 1). The percentage of time spent feeding increased to 2.25–2.49% from 7 to 18 h after placement. During the last 6 h of the day, the percentage of time spent feeding increased significantly, to 21.77 ± 0.57% (Figure 1).

Poults were observed drinking 2.49 ± 0.06% of time. There was no effect of biological age [*F*_(1, 19)_ = 0.09; *P* = 0.76] or dietary [*F*_(2, 17)_ = 0.10; *P* = 0.90] treatments, nor an interaction between the two factors [*F*_(2, 22)_ = 0.22; *P* = 0.80]. Drinking behavior was significantly affected by time [*F*_(3, 28)_ = 19.71; *P* < 0.0001). Drinking behavior peaked in the first 6 h after placement, declined through the next 6 h and then gradually increased over the next 12 h (Figure 1).

Poults were active 12.30 ± 0.25% of time over the first 24 h after placement. There was no effect of biological age [F_(1, 21)_ = 0.34; *P* = 0.56], diet [*F*_(2, 19)_ = 1.52; *P* = 0.24], nor an interaction between the two factors [*F*_(2, 20)_ = 1.39; *P* = 0.27] on time spent active. Time spent active was significantly influenced by time period [F_(3, 35)_ = 85.46; *P* < 0.0001]. Poults were quite active upon first placement, with 26.21 ± 0.73% of the time active during the first 6 h. However, over the next 18 h after placement, active behavior decreased to 7.71 ± 0.29% of time (Figure 1).

### Latency to Feed and Drink

Mean latencies to feed and drink were 823.75 ± 193.02 s (13.73 min) and 348.63 ± 169.79 s (5.81 min), respectively. There were no differences between biological ages, dietary treatments nor an interaction between the two (Table 2).

**Table 2:** Latencies to feed and drink (LS means ± SEM). Neither biological age, diet nor the interaction between the two influenced latency to eat or drink.

**Treatment**	**Latency to feed, s**	**Latency to drink, s**
**BIOLOGICAL AGE**		
Early	659.23 ± 186.52	512.00 ± 311.20
Standard	1018.18 ± 361.44	155.55 ± 20.92
**DIET**		
Control	603.13 ± 168.27	653.38 ± 504.08
A	747.50 ± 258.39	182.75 ± 28.38
B	1120.63 ± 501.78	209.75 ± 96.48
***P*****-VALUES**		
Biological age	*F*_(1, 15.2)_ = 0.85; *P* = 0.37	*F*_(1, 15.7)_ = 0.59; *P* = 0.46
Diet	*F*_(2, 12.2)_ = 0.33; *P* = 0.73	*F*_(2, 11.9)_ = 0.58; *P* = 0.57
Age × Diet	*F*_(2, 16.5)_ = 1.30; *P* = 0.30	*F*_(2, 17.7)_ = 1.38; *P* = 0.28

### Behavior Through Day 5

Time affected the behavior of turkey poults. While patterns of inactivity increased with time, percentage of poults at the feeder decreased with time (Table 3). Drinking and active behavior, did not fluctuate with time, and took up <10% of the time budget. There was no effect of biological age, diet, nor an interaction between the two on the percentage of time poults spent performing any behavior patterns through 5 d of age (Table 3).

**Table 3:** Percentage of time (LS mean ± SE) poults spent feeding, drinking, active or inactive on days 2, 3 and 5 after placement.

**Day**	**Feeding, %**	**Drinking, %**	**Active, %**	**Inactive, %**
2	26.30 ± 1.59	3.16 ± 0.17	6.69 ± 0.45	63.85 ± 1.50
3	19.24 ± 0.67	3.86 ± 0.17	6.27 ± 0.38	70.62 ± 0.67
5	7.64 ± 0.29	4.50 ± 0.22	8.12 ± 0.49	79.74 ± 0.62
***P*****-VALUES**				
Biological age	*F*_(1, 119)_ = 0.29; *P* = 0.59	*F*_(1, 119)_ = 1.19; *P* = 0.28	*F*_(1, 119)_ = 0.17; *P* = 0.68	*F*_(1, 119)_ = 0.99; *P* = 0.32
Diet	*F*_(2, 119)_ = 0.02; *P* = 0.98	*F*_(2, 119)_ = 1.08; *P* = 0.34	*F*_(2, 119)_ = 0.28; *P* = 0.76	*F*_(2, 119)_ = 0.33; *P* = 0.72
Age × diet	*F*_(2, 119)_ = 0.08; *P* = 0.92	*F*_(2, 119)_ = 0.81; *P* = 0.45	*F*_(2, 119)_ = 0.39; *P* = 0.68	*F*_(2, 119)_ = 0.06; *P* = 0.94

### Productivity

**Figure 2:**
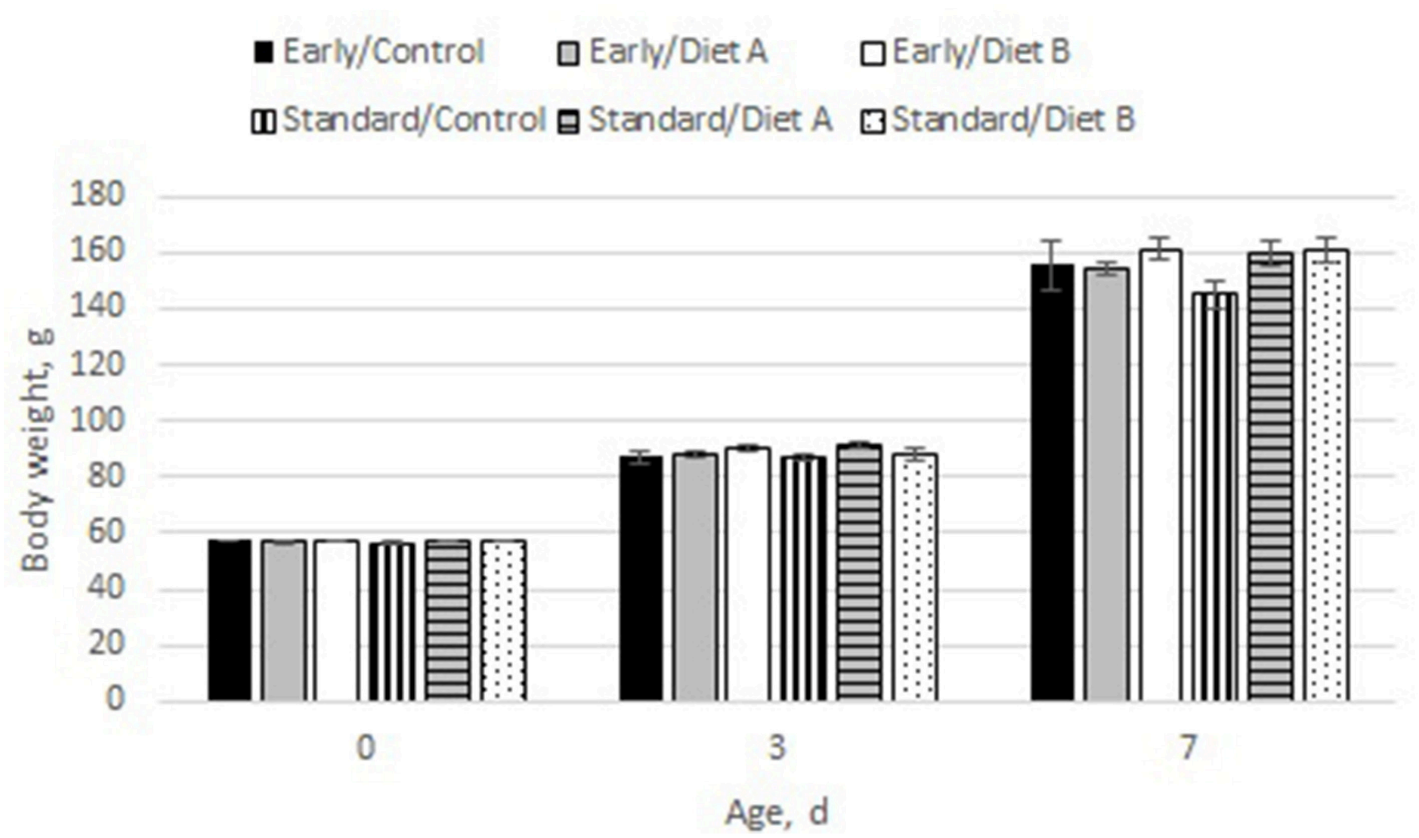
Changes in body weights by biological age and diet. There was an interaction between biological age and diet by day [*F*_(4,19)_ = 2.99; *P* = 0.046], with standard hatch poults fed the Modified B diet weighing the least at 7 d of age.

Poults weighed 56.97 ± 0.14 g at placement (range: 40.2–70.0 g), 88.65 ± 0.29 g on day 3 (range: 45.3–111.7 g) and 156.32 ± 0.61 g on day 7 (range: 96.4–205.74 g). Although there was no effect of biological age [*F*_(1, 18)_ = 0.14; *P* = 0.71] or diet [*F*_(2, 18)_ = 2.07; *P* = 0.16] on body weight, there was an interaction between biological age, diet and day [*F*_(4, 19)_ = 2.99; *P* = 0.046]. Treatments had similar body weights at d 0 and d 3. However, on d 7, standard hatch poults fed the control diet weighed less than all other poults (Figure 2).

Average daily gain (ADG) over the course of 7 d was 13.57 ± 0.30 g/d. There was no effect of biological age on ADG [*F*_(1, 15)_ = 0.00; *P* = 0.95] but a trend toward an effect of diet [*F*_(2, 15)_ = 3.45; *P* = 0.059]. Poults on the control diet gained 12.69 ± 0.57 g/d, whereas those on the Modified A diet gained 13.71 ± 0.57 g/d and those on the Modified B diet gained 14.25 g/d. There was no interaction between biological age and diet on ADG [*F*_(2, 15)_ = 1.40; *P* = 0.28]. Similarly, the feed conversion ratio (FCR) was not influenced by biological age [*F*_(1, 18)_ = 0.19; *P* = 0.67], but there was a tendency for an effect of diet [*F*_(2, 18)_ = 2.64; *P* = 0.099]. Poults on the control diet had an FCR of 1.28 ± 0.03 g FI /g BW. Poults on the Modified B diet were the most efficient, with an FCR of 1.19 ± 0.03 g/g, whereas those on the Modified A diet were intermediate with an FCR of 1.21 ± 0.03 g/g. There was no interaction between biological age and diet on FCR [*F*_(2, 18)_ = 1.57; *P* = 0.24].

### Flips and Mortality

Overall, 1.88% of turkey poults flipped (18 out of 960). Three of these poults flipped more than once, and seven of the poults that flipped were culled or died. There was no effect of biological age [*F*_(1, 16)_ = 1.08; *P* = 0.31], diet [*F*_(2, 12)_ = 0.15; *P* = 0.86], nor an interaction between the two [*F*_(2, 18)_ = 0.20; *P* = 0.82] on the percentage of poults that flipped (Table 4).

**Table 4:** Effect of biological age and Ca:P ratio in the diet on flips, starve outs and yolk sac infections (LS Means ± SEM).

**Treatment**	**Flips, %**	**Starve-outs, %**	**Yolk sac, %**
Early hatch	1.39 ± 0.97	0.63 ± 0.33	3.61 ± 0.72
Standard hatch	2.55 ± 1.04	1.81 ± 0.35	1.56 ± 0.77
Control diet	1.56 ± 1.10	0.94 ± 0.41	2.81 ± 0.85
Modified A	2.19 ± 1.10	1.25 ± 0.41	2.50 ± 0.85
Modified B	2.14 ± 1.14	1.46 ± 0.43	2.43 ± 0.88
**EARLY HATCH**			
Control diet	0.98 ± 0.87	0.63 ± 0.58	4.99 ± 1.88
Modified A	1.43 ± 1.15	1.25 ± 0.58	3.70 ± 1.59
Modified B	0.85 ± 0.72	1.25 ± 0.58	2.01 ± 1.04
**STANDARD HATCH**			
Control diet	1.43 ± 1.51	1.25 ± 0.58	0.62 ± 0.62
Modified A	1.97 ± 1.43	0.00 ± 0.52	1.25 ± 0.90
Modified B	2.29 ± 1.79	2.92 ± 0.67	2.83 ± 1.57
***P*****-VALUES**			
Biological age	*F*_(1, 16)_ = 1.08; *P* = 0.31	*F*_(1, 12)_ = 6.02; *P* = 0.03	*F*_(1, 15)_ = 4.92; *P* = 0.04
Diet	*F*_(2, 12)_ = 0.15; *P* = 0.86	*F*_(2, 12)_ = 0.39; *P* = 0.68	*F*_(2, 12)_ = 0.07; *P* = 0.93
Age × Diet	*F*_(2, 18)_ = 0.20; *P* = 0.82	*F*_(2, 12)_ = 3.33; *P* = 0.07	*F*_(2, 17)_ = 2.73; *P* = 0.094

**Figure 3:**
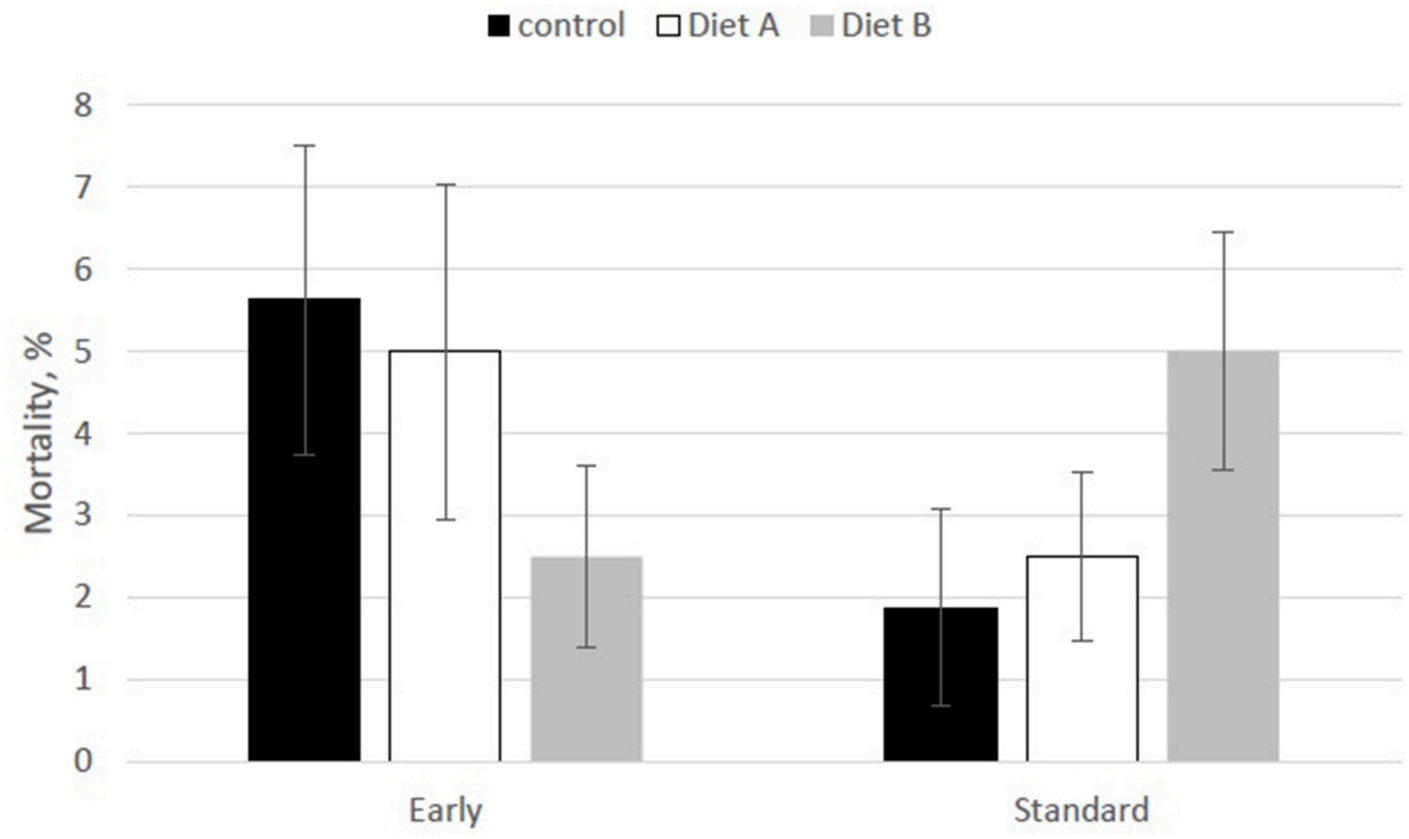
Effect of biological age and diet on early mortality in turkey poults. There was an interaction between biological age and diet [*F*_(2, 18)_ = 3.65, *P* = 0.047]; early hatch poults had the lowest mortality rate on the modified B diet, whereas standard hatch poults experienced the highest mortality rate on this diet.

The overall mortality rate for the 7 d trial was 4.07% (39/960 poults). Most of the mortality recorded (76.9%) occurred between d3 and 7. Cause of death for 59.3% of the mortalities was a yolk sac infection. Starve-outs accounted for 32.3% of mortalities and unknown causes accounted for the remaining losses. There were no significant biological age [*F*_(1, 15)_ = 0.82; *P* = 0.38] or diet [*F*_(2, 12)_ = 0.07; *P* = 0.93] effects on overall mortality. However, there was an interaction between biological age and diet [*F*_(2, 16)_ = 4.01; *P* = 0.038]; early hatch poults had the lowest mortality rate (2.50 ± 1.16%) on the modified B diet, whereas standard hatch poults experienced the highest mortality rate on this diet (6.67 ± 2.43%; Figure 3).

Standard hatch poults had a greater percentage of starve-outs (1.70 ± 0.42%) than early hatch poults [0.58 ± 0.30%; *F*_(1, 12)_ = 6.02; *P* = 0.03), although there was a trend toward an interaction with diet [*F*_(2, 12)_ = 3.33; *P* = 0.07; Table 4]. Standard hatch poults on the modified B diet had the highest percentage of starve outs, whereas those on the modified A diet had the lowest. Biological age also influenced the percentage of yolk sac infections, although in the opposite direction to starve-outs [early hatch: 3.46 ± 0.78%; standard hatch: 1.48 ± 0.60%; *F*_(1, 15)_ = 4.92; *P* = 0.04]. There was no effect of diet but a tendency for an interaction between diet and biological age [*F*_(2, 17)_ = 2.73; *P* = 0.094; Table 4].

## Discussion

The objective of this study was to examine the relationships among biological age, diet, the development of poult feeding behavior and early mortality. Although there were no differences among biological age groups or dietary treatments in behavior over the first 24 h after placement, time played a significant role for all measured behavior. To our knowledge, this has not been previously quantified in poults and it provides a basis for further exploration of early time budgets in turkeys. Within 6 h of placement, poults shifted from active to resting and continued to rest for approximately 75% of the first 24 h after placement. This amount of rest is higher than what has been observed in broiler chickens [39–58% of time spent resting during first 24 h; (19)], although it may have been influenced by the 24 h light period upon placement, which was found to increase lethargy in older turkeys (20). This observation may indicate the need for dark periods or longer dark periods during the first days after placement, when no or minimal dark is typically provided. After the initial rest period, feeding behavior increased, probably as a function of increasing motivation [e.g., (21)] and in part due to social facilitation (22).

Latencies to feed and drink were short and in agreement with previous reports for newly placed poults (8, 23). There were no differences between biological age or dietary treatments. We had expected a difference between the biological age treatments, as Nielsen et al. (24) reported more early-hatched broiler chicks feeding during the first 48 h compared to middle- or late-hatched chicks. The lack of difference may be due to the actual differences in age between the early and standard hatched groups. While the early hatch group was selected the day prior to placement to ensure they were at least 30 h old at placement, the standard hatch was heterogeneous and may have included poults that hatched soon after the early hatch poults. Because we used a commercial hatchery, we were not able to identify or pull poults more frequently, or determine the actual difference in biological age between the two treatments, as has been done in other experiments (3, 18, 24, 25). We also did not observe an effect of diet on latency to feed and drink. The lack of effect could indicate that the motivation to investigate potential feed sources was the same for poults regardless of dietary treatment. While the diets differed in their percent of fine particles and mineral concentrations, we did not anticipate that this difference would influence their latency to feed, although published reports have only examined the effect of fines on older turkey performance (11, 14).

Performance variables were not affected by biological age directly, but there was an interaction between biological age and dietary treatment. Standard hatch poults on the control diet had the lowest body weight at day 7. All poults on the control diet tended to have lower average daily gain and worse feed efficiency than poults on the other two diets. Standard hatch poults on the control diet also had the lowest mortality. Lower body weight, average daily gain and worse feed efficiency may have been skewed due to low mortality in the standard hatch group fed the control diet. It's unclear what, specifically, about the control diet may have contributed to these differences. The control diet had higher percentage of fines compared to the other diets, which may have reduced performance (11, 14). It also had the lowest inclusion of calcium. Sanders et al. (12) determined that optimum growth through 7 days of age occurred with 9.6 g/kg calcium inclusion, which supports another study that reported calcium concentrations below 10 g/kg to be a limiting factor in body weight growth from 4 to 7 weeks of age (26). Because nutrient concentrations and feed quality were confounded between diets, we cannot determine which aspect of the control diet contributed to the worse productivity. In addition, we did not follow poults beyond 7 days of age, so we do not know if remaining small poults in the control treatment would have died or necessitated culling, which would influence both growth and mortality data.

Poults fed the Modified B diet had the highest percentage of mortality due to starvation. While the Modified B diet had intermediate levels of both Ca and P and proportion of fines compared to the other two diets, it had a lower calcium to phosphorus ratio than either the control or Modified A diet. Sanders and colleagues (12) reported that a Ca:P ratio of 1.25 or greater is needed for optimum growth in turkey poults but, to the authors' knowledge, there is no published literature on minimum Ca:P ratios to optimize feeding behavior and reduced mortality. Further research is needed to understand the interaction between specific nutrients and diet quality on mortality, as other differences in the diets may have led to differences in glucose metabolism, which has been suggested to influence poult mortality (4, 10, 27).

Flips or flip-overs have been described as weak poults that fall over and are unable to right themselves (2, 28). Bate (5) suggested that flips are the real cause of starvation, as poults unable to right themselves would be unable to feed. Christensen et al. (2), however, found that poults that had flipped all had feed in their digestive tract. There are few reports on the prevalence of flips, but the prevalence flipped in our experiment (1.88%) is in line with Noble et al. (28) found in a randombred line. While Nielsen et al. (24) reported that late hatched slow growing broilers had greater mortality due to flips than early hatched broilers, we found no effect of biological age or diet on flips.

Our finding that the majority of poults that died had consumed feed is contrary to the idea that starvation is the main cause of early mortality (2, 4). Yet, >1% of placed poults died due to starvation under these experimental conditions, which still represents a significant welfare concern. While the overall mortality rate in the current experiment (4%) was in line with some reports (3, 4, 23), it was considerably higher than was reported for commercial flocks (1.5–2.2%; 3). Biological age treatment had a significant effect on the cause of death, with earlier hatched poults dying more from yolk sac infections and less from starvation than standard hatch poults. Mortalities in the early hatch treatment were primarily due to yolk saculitis, a bacterial infection. Bacteria is commonly found in the yolk sac of poults (29), which may colonize and cause infections. Earlier hatched birds may be more prone to bacterial infections due to their extended exposure to pathogens in the hatchery and increased potential of becoming dehydrated (17). In the standard hatch group, mortality occurred in equal parts due to yolk sac infections and starvation, although the mortality was primarily due to those fed on the Modified B diet. This finding is in agreement with previous researchers who suggested that biological age and diet composition interact on poults' metabolic system, decreasing feed intake and leading to starve-outs (4, 10).

In conclusion, biological age and diet did not influence the development of feeding behavior in the current experiment. While lack of feeding behavior or lack of feed consumption is assumed to be a major cause for early poult mortality, yolk sac infections were the leading cause of mortality under our experimental conditions. Biological age and diet interacted to differentially influence growth and mortality, with standard hatch poults fed the diet with the greatest fines and lowest calcium concentration experiencing the slowest growth and those fed the diet with the lowest Ca:P experiencing the highest mortality.

## Ethics Statement

This study was approved by the Nutreco Canada AgResearch Animal Care Committee, and conducted at the Shur-Gain Research Facility, Burford, Ontario in 2013.

## Author Contributions

CR and ST conceived the study and analyzed the data and co-wrote the paper. The experimental procedures were designed and performed by CR.

### Conflict of Interest Statement

At the time of the study, CR was employed by Nutreco Canada Agresearch, where the study occurred. The remaining author declares that the research was conducted in the absence of any commercial or financial relationships that could be construed as a potential conflict of interest.
